# Tenodesis of the Long Head of the Biceps Tendon Has No Detrimental Impact on the Postoperative Outcome After Supraspinatus Tendon Reconstruction

**DOI:** 10.7759/cureus.71976

**Published:** 2024-10-20

**Authors:** Mirco Sgroi, Thomas Caffard, Marius Ludwig, Thomas Kappe, Heiko Reichel

**Affiliations:** 1 Department of Orthopedic Surgery, University of Ulm, Ulm, DEU

**Keywords:** fatty infiltration, lhb, repair, rotator cuff tear, tenodesis

## Abstract

Introduction

While several studies have compared tenotomy and tenodesis, few studies have examined whether performing a tenodesis of the long head of the biceps (LHB), when indicated, in patients who have undergone rotator cuff reconstruction has a detrimental impact on clinical and radiological postoperative outcomes. The present study aimed to investigate whether performing a tenodesis of the LHB has a damaging effect on the clinical and radiological outcome after rotator cuff reconstruction.

Material and methods

Fifty-one patients surgically treated for supraspinatus (SSP) tendon tears were included. All included patients received a reconstruction of the SSP, depending on the LHB surgery performed, patients were divided into two groups: 1) with concomitant tenodesis of the LHB and 2) without surgery of the LHB. Western Ontario Rotator Cuff Index (WORC), American Shoulder and Elbow Surgeons Shoulder Score (ASES), Constant, Oxford, and LHB scores were assessed at 2.3 ± 0.3 years postoperatively. All patients underwent clinical examination, including range of motion and force measurements. Furthermore, patients received an MRI scan of the operated shoulder two years postoperatively. Two blinded observers independently analyzed the integrity and quality of the rotator cuff on postoperative MRI using Sugaya and Castricini classifications. Clinical and radiological results were compared between both groups.

Results

All analyzed clinical scores, ranges of motion, and force measurements revealed no difference between both groups. Except for fatty infiltration (0° = 81% (21 of 26) vs. 68% (17 of 25); 1° = 15% (four of 26) vs. 28% (seven of 25); 2° = 4% (one of 26) vs. 4% (one of 25); and 3° = 0% (0 of 26) vs. 4% (one of 25); p < 0.01), no differences between both groups concerning the integrity (re-rupture rate = 27% (seven of 26) vs. 20% (five of 25); p = 0.39) and quality of the SSP tendon were found.

Conclusions

Tenodesis of the LHB performed in addition to rotator cuff repair is not associated with detrimental clinical outcomes than rotator cuff repair without surgery of the LHB. Except for fatty infiltration, which was lower in the tenodesis group, the results of the present study suggest that concomitant tenodesis of the LHB produces in patients who received rotator cuff repair have no detrimental effect in terms of clinical outcomes and re-rupture rates as well as tendon quality. Therefore, when indicated, simultaneous tenodesis of the LHB appears to be a safe and effective procedure that has no negative impact on the postoperative outcome after SSP tendon reconstruction.

## Introduction

The long head of the biceps tendon (LHB) plays a critical role in the pathogenesis of rotator cuff tears [1,2]. Pathologies of the LHB have been identified as one of the most important causes of shoulder pain [3]. Instability, partial rupture, and SLAP lesions are the most common pathologies of the LHB [4]. Rupture of the pulley complex causes instability of the LHB, which can be associated with concurrent rotator cuff lesions because of its anatomic proximity to the rotator cuff [4,5]. Surgical intervention on the LHB should therefore often supplement rotator cuff repair [6]. The LHB exerts a caudalizing effect on the humeral head so that surgery of the LHB can influence the healing of the rotator cuff. Surgical treatment of the LHB essentially involves tenotomy or tenodesis [7-9]. Many studies have compared tenotomy and tenodesis with controversial results [10-12]. Various proposed tenodesis techniques have been investigated with discordant results [9,13,14]. However, just a few studies have examined whether, performing a tenodesis of the LHB, when indicated, in patients who have undergone rotator cuff reconstruction has a detrimental impact on clinical and radiological postoperative outcomes [15]. Furthermore, it is not known whether the lack of a caudalizing effect of the LHB on the humeral head in patients who have undergone tenodesis of the LHB can lead to additional mechanical stress and thus to an increased rate of re-rupture of the reconstructed rotator cuff. Therefore, the following research questions were investigated: (1) Does performing an additional tenodesis of the LHB influence the postoperative course after a rotator cuff repair? (2) Do differences in re-rupture rates and tendon quality occur between patients with rotator cuff repair without LHB tenodesis and those with concomitant LHB tenodesis? The null hypothesis was that patients with concomitant tenodesis of the LHB would have a delayed and poorer clinical outcome as well as a higher re-rupture rate compared to patients without tenodesis of the LHB.

## Materials and methods

Study design and setting

This study examined the impact of LHB tenodesis on postoperative clinical and radiographic outcomes two years after rotator cuff repair. It was performed at one center in Germany and involved two observers, who were residents (T. C. and M. L.) in orthopedics at the researchers’ clinic. Independent institutional review board approval was obtained from the ethics committee of the University of Ulm (number 104/17).

Participants

Sixty-three patients treated surgically for supraspinatus (SSP) tendon tears in the researchers’ department were checked for inclusion in the present study. Of these 8% (five of 63) were excluded because they had a spontaneous rupture of the LHB at the time of surgery or had undergone a tenotomy of the LHB. Another 11% (seven of 63) were excluded because planes or slices (e.g., sagittal, axial, or frontal) of MRI were missing in whole or in part, which left 81% (n = 51) for this analysis. All included patients had a minimum follow-up of two years; the mean follow-up was 2.3 ± 0.3 years postoperatively.

Preoperative radiologic setup

As part of the preoperative planning and given clinical requirements, MRI was preoperatively and independently performed on all shoulders involved in this study. MR images were obtained with a 1.5 Tesla MRI scanner without contrast medium application.

Surgical technique

One experienced shoulder surgeon (T. K.) who was not involved in the radiologic examinations, performed the surgeries. The surgical indication was based on patient history, clinical examination, and MRI findings. All patients had clinically relevant rotator cuff tears and were examined preoperatively using MRI. For all patients, the decision regarding surgery was made independently of this study. All arthroscopies were performed with the patient under brachial plexus block and general anesthesia. Patients were positioned in the beach chair position, and an arm-holding device (Trimano, Maquet) was used to hold the operated arm. Posterior, anterolateral, and lateral portals served as standard approaches. Diagnostic arthroscopy was performed. All relevant pathologies identified during surgery were treated. 

The indication for tenodesis of the LHB was determined based on the preoperative MRI and was confirmed by the intraoperative findings. Tenodesis of the LHB was performed in case of instability of the LHB (Pulley lesions), SLAP lesions (> 1°), or partial tears. All tenodesis of the LHB were performed using one technique: LHB was tenotomized, passed extracorporeally, and secured with a Fiberwire. Afterward, tenodesis of the LHB was performed with a 5.5 mm biocomposite SwiveLock (Arthex, Naples, Fl, USA) in the proximal part of the biceps groove, whereby the end of the LHB was locked in the bone tunnel with the SwiveLock.

Rehabilitation

After the surgical procedure, the affected arm was immobilized in an abduction pillow (Ultra Sling III, Donjoy, USA) for six weeks. Patients were allowed to perform passive exercises for six weeks. Afterward, the pillow was discarded, and active exercises without weight-bearing were initiated. Strengthening exercises were recommended after 13 weeks.

Clinical assessment at follow-up

The surgeon was not involved in the clinical or radiological follow-up examinations. Depending on the received surgical treatment, patients were retrospectively divided into two groups: a group of patients who underwent rotator cuff repair without LHB surgery and a second group of patients who underwent rotator cuff repair with simultaneous tenodesis of the LHB. The indication for tenodesis of the LHB was based on the preoperative MRI examination and confirmed intraoperatively, taking into account the following criteria: Instability of the LHB (pulley lesion), SLAP lesions (> 1°), or partial tears. A resident in sports orthopedics (D. K.) conducted a clinical assessment of all patients at 2.3 ± 0.3 years follow-up. The patients were asked to complete the following four clinical shoulder scores: American Shoulder and Elbow Surgeons Shoulder Score (ASES) [16], Constant Shoulder Score (CS) [17] Western Ontario Rotator Cuff Index (WORC) [18], and Oxford Shoulder Score (OSS) [19]. Patients who had undergone tenodesis of the LHB completed a specific LHB score. Scheibel et al. introduced the LHB score, which comprises three parts: pain/cramps (max. 50 points), cosmesis (max. 30 points), and elbow flexion strength (max. 20 points). The best possible result is 100 points, while the worst possible result is 0 points [20]. All patients underwent a clinical examination that included measuring range of motion and specific shoulder tests. The specific clinical tests performed were the Jobe full can and empty can tests.

Radiological examination at follow-up

An MRI was performed at the time of follow-up (2.3 ± 0.3 years) using a 1.5 Tesla MRI scanner (MAGNETOM TIM-Symphony, Siemens, Erlangen, Germany). Patients were positioned supine with the arm in neutral rotation by the side of the body. A dedicated standard shoulder coil was placed over the shoulder. Two blinded residents in orthopedics (T. C. and M. L.) performed the analysis of the MRI scans, which included measurements from preoperative and postoperative MRI. To obtain intra- and interrater correlations, both observers performed all measurements independently; one of them, blinded to the first measurements, took repeated measurements after at least six weeks. All measurements resulted in excellent intra-rater (Spearman-Rho = 0.97, CI 95% 0.96-0.99; p > 0.01) and inter-rater (ICC = 0.96, CI 95% 0.96-0.97; p> 0.01) reliabilities. To investigate the quality and morphological integrity of the SSP tendon, the blinded researchers determined the following radiological parameters using the postoperative MRI scans: Sugaya classification [21-23], SSP tendon integrity according to Castricini et al. [24] (including signal intensity, footprint coverage, and tendon thickness), fatty infiltration of the SSP muscle according to Goutailler [25], atrophy of the SSP muscle according to Thomazeu [26].

Statistical analysis

On the basis of previously published studies, a power analysis was performed for the outcome scores. This analysis determined that for a significance level of 5%, a sample size of 51 patients was sufficient to provide a power of 0.82. The statistical analysis was performed using SPSS (version 24, IBM Corp., Armonk, NY). All collected data were secured in a computerized database. Descriptive baseline statistics were used. In addition, patients were divided into two groups depending on whether LHB surgery was performed or not. When comparing clinical and radiological results between both groups, a one-sample t-test for interval-scaled, the Wilcoxon test for ordinal-scaled, and the chi-square test for nominal-scaled parameters were used. To obtain intra- and interrater correlations, two orthopedic surgeons repeated all measurements; one of them, blinded to the first measurements, took repeated measurements after at least six weeks. The intra-rater reliability was evaluated with the Spearman-Rho coefficient for ordinal-scaled measurements. The inter-rater reliability was calculated using the intraclass correlation coefficient (ICC). Intra-rater correlation coefficients are generally interpreted as follows: < 0.5 = poor, 0.5-0.75 = moderate, 0.75-0.9 = good, and > 0.9 = excellent. Differences for p-values < 0.05 were considered significant.

## Results

Descriptive data

About 51% (26 of 51) were women. The patients’ mean age was 66 ± 10 years. The right side was affected in 71% (36 of 51) of the patients. The average BMI was 29 ± 5. Most patients (71%; 36 of 51) were classified as Grade 1 or 2 according to the American Society of Anesthesiologists (ASA) classification. No difference in baseline data between patients who had and had not received concomitant LHB tenodesis was found (Table 1).

**Table 1:** Preoperative demographic and radiologic data of the investigated patients BMI = body mass index; ASA = American Society of Anesthesiologists classification; SD = standard deviation; interval-scaled variables were tested with the t-test. Significance level = < 0.05.

Parameters	Tenodesis						No tenodesis						P-value
Analyzed patients (n)	26						25						
Women, % (n)	54 (14)						48(12)						0.71
Side involved, right % (n)	69 (18)						72 (18)						0.16
Age in years, mean ± SD	65 ± 8						68 ± 11						0.24
ASA classification, % (n)	ASA 1	ASA 2		ASA 3		ASA 4	ASA 1	ASA 2		ASA 3		ASA 4	0.59
	4 (1)	73 (19)		23 (6)		0 (0)	8 (2)	58 (15)		35 (9)		0 (0)	
BMI, mean ± SD	28 ± 5						30 ± 4						0.22
Atrophy SSP, % (n)	1°		2°		3°		1°			2°	3°		0.15
	88 (23)		11 (3)		0 (0)		69 (18)			27 (7)	4 (1)		
Fatty Infiltration SSP, % (n)	0°	1°	2°		3°		0°		1°	2°	3°		0.83
	69 (18)	23 (6)	4 (1)		4 (1)		92 (24)		4 (1)	4 (1)	0 (0)		
Upward Migration Index, ratio	1.32 ± 0.09						1.27 ± 0.9						0.73

Concomitant pathologies

No differences between both groups concerning concomitant pathologies were found. Results are presented in Table 2. 

**Table 2:** Additional treatments performed on both groups. SD = standard deviation; LCR = lateral clavicula resection; SAD = subacromial decompression; interval-scaled variables were tested with the t-test. Significance level = < 0.05.

Treatment	No tenodesis (n = 26)	Tenodesis (n =25)	P-value
SSC repair, % (n)	42 (11)	69 (18)	0.60
LCR, % (n)	11 (3)	27 (7)	0.26
SAD, % (n)	73 (19)	85 (22)	0.79

Clinical comparison between patients with and without tenodesis of the LHB

No difference was found for any of the scores used between patients who received LHB and patients without surgery of the LHB. Results are presented in Table 3.

**Table 3:** Comparison between patients with and without tenodesis of the LHB in relation to the clinical outcome AS = acromial slope. CI = confidence interval.

Variable	Tenodesis (n = 26)	No tenodesis (n = 25)	Difference (95% CI)	P-value
WORC, points ± SD	98 ± 2	97 ± 2	1 (-0.4– 2)	0.19
Constant, points ± SD	70 ± 18	72 ± 14	2 (-7 – 11)	0.73
Oxford, points ± SD	25.3 ± 12	24.8 ± 13	0.5 (-6.80–7.97)	0.87
ASES, points ± SD	78 ± 21	85 ± 16	7 (-4 – 17)	0.22
LHB, points ± SD	84 ± 14	none	none	-

No differences between both groups concerning the range of motion. Results are presented in Table 4.

**Table 4:** Range of motion and Jobe test of the tenodesis and no tenodesis groups ROM = range of motion; SD = standard deviation; interval-scaled variables were tested with the t-test. Significance level = < 0.05.

ROM	Tenodesis (n =26)	No tenodesis (n = 25)	Difference (95% CI)	P-value
Abduction (° ± SD)	165 ± 27	162.00 ± 37	3 (-15 - 21)	0.71
Adduction (° ± SD)	38.4 ± 15.6	38.0 ± 19.5	0.4 (-9.5 - 10.4)	0.92
Flexion (° ± SD)	165 ± 28	161 ± 41	4 (-15 - 24)	0.65
Extension (° ± SD)	41 ± 16	46± 15	5 (-13 - 4)	0.28
External rotation low (° ± SD)	62 ± 22	58 ± 21	4 (-5 - 13)	0.39
External rotation high (° ± SD)	73 ± 23	80 ± 19	7 (-9 - 15)	0.61
Internal rotation high (° ± SD)	61 ± 22	58 ± 21	7 (-5 - 19)	0.25
Jobe Test Full Can % (n) positive	11 (3)	15 (4)	4 (1)	0.43
Jobe Test Empty Can % (n) positive	19 (5)	11 (3)	8 (2)	0.37

No difference between patients with and without tenodesis concerning force in different starting positions was observed. The results concerning force measurement are presented in Table 5.

**Table 5:** Force measurement in both groups in different initial positions of the affected arm SD = standard deviation; interval-scaled variables were tested with the t-test. lb = pound-force; interval-scaled variables were tested with the t-test. Significance level = < 0.05.

Arm starting position	No tenodesis (n = 26)	Tenodesis (n = 25)	Difference (95% CI)	P-value
0° abduction (lb ± SD)	15.4 ± 7	15.5 ± 10	0.1 (-5- 2)	0.96
90° abduction, external rotation (lb ± SD)	10 ± 4	8 ± 4	2 (-1 - 4)	0.22
90° abduction, internal rotation (lb ± SD)	9 ± 4	7 ± 4	2 (-1 - 0.8)	0.18
Low external rotation (lb ± SD)	13 ± 6	12 ± 5	1 (-2 - 4)	0.60
High external rotation (lb ± SD)	7 ± 3	6 ± 3	1 (-1 - 2)	0.57

Radiological comparison between patients with and without tenodesis of the LHB

Except for fatty infiltration (0° = 81% (21 of 26) vs. 68% (17 of 25); 1° = 15% (four of 26) vs. 28% (seven of 25); 2° = 4% (one of 26) vs. 4% (one of 25); and 3°= 0% (0 of 26) vs. 4% (one of 25); p < 0.01), no difference between the tenodesis and LHB surgery groups concerning the integrity (re-rupture rate = 27% (seven of 26) vs. 20% (five of 25); p = 0.39) and quality of the rotator cuff was found (Table 6).

**Table 6:** Clinical and radiological outcomes of both analyzed groups CI = confidence interval.

Variable	Tenodesis (n = 26)				No tenodesis (n = 25)				P-value
Re-Rupture rate, % (n)	27 (7)				20 (5)				0.39
Tendon thickness, % (n)	1°		2°	3°	1°	2°	3°		
	23 (6)		35 (9)	42 (11)	36 (9)	44 (11)	24 (6)		0.11
Footprint coverage, % (n)	11 (3)		8 (2)	81 (21)	24 (6)	20 (5)	60 (15)		0.07
Tendon quality, % (n)	19 (5)		15 (4)	65 (17)	32 (8)	32 (8)	40 (10)		0.06
Muscle atrophy, % (n)	92 ((24)		2 (7.7)	0 (0)	64 (16)	40 (10)	0 (0)		0.18
Upward migration, ratio ± SD	1.33 ± 0.09				1.27 ± 0.10				0.69
Fatty infiltration, % (n)	0°	1°	2°	3°	0°	1°	2°	3°	
	81 (21)	15 (4)	4 (1)	0 (0)	68 (17)	28 (7)	4 (1)	4 (1)	p < 0.01

## Discussion

The most important finding of this study was that performing additional LHB tenodesis has no detrimental effect on clinical outcomes in patients who received rotator cuff repair. Furthermore, except for fatty infiltration, which was lower in the tenodesis group, no difference between both groups regarding re-rupture rate or tendon quality was found. LHB tenodesis seems to not lead to inferior clinical or radiological results. Future researchers should verify these results, considering a longer follow-up period, and including different LHB tenodesis techniques.

Comparison of clinical outcomes after rotator cuff repair with LHB tenodesis and without LHB surgery

The present study showed that patients receiving tenodesis of the LHB had no detrimental clinical outcomes in terms of quality of life, range of motion, and strength compared with rotator cuff repair without LHB surgery. This finding is important because LHB tenodesis is often performed in rotator cuff repair, but its effects on postoperative clinical outcomes are not fully understood. Several studies have evaluated the postoperative outcome in patients after surgical treatment of the LHB [11,27,28]. Most of these studies have compared tenotomy with tenodesis or various tenodesis techniques. De Carli et al. [28] found no difference between tenotomy and tenodesis regarding clinical and functional outcomes, except for a more pronounced occurrence of Popeye’s sign in the tenotomy group, which, however, had no functional consequences. In another study, tenodesis and tenotomy indicated improvement in functional outcomes, but three times higher Popeye deformity was observed in the tenotomy group [29]. Watson et al. conducted the only study comparing rotator cuff repair without surgery of the LHB against patients with concomitant LHB tenodesis [15]. In this study, which analyzed 80 patients one year after rotator cuff repair, patients with concurrent LHB tenodesis had greater clinical improvement and a higher ASES score. A detailed comparison between the present study and the studies mentioned above is not possible due to the different settings. Conversely, the current study does not confirm Watson et al.’s results [15]. This is because the current study did not find a better outcome in patients who received rotator cuff repair with concurrent LHB tenodesis, as reported by Watson et al. Nevertheless, it worth noting is that this study had a longer follow-up period than Watson et al.’s. In the researchers’ clinical experience, especially for the LHB, the duration of follow-up seems to play a major role. Future research should investigate whether this study’s results are confirmed when a different technique, such as subpectoral tenodesis, is used for tenodesis of the LHB.

Comparison of radiological outcomes after rotator cuff repair with and without LHB tenodesis

Apart from fatty infiltration, which revealed better results in patients with concurrent LHB tenodesis, the researchers found no difference between patients with rotator cuff repair without LHB surgery and those with concomitant LHB tenodesis concerning re-rupture rate and tendon quality. These results are interesting because they provide new insights into the relationship between LHB tendon and rotator cuff healing after repair. This issue is crucial because the LHB tendon plays an important biomechanical role for the rotator cuff and therefore performing tenodesis can alter these biomechanical conditions and appears to be associated with healing of the SSP tendon. Performing an LHB tenodesis seems to be not associated with a higher rate of SSP rupture or inferior tendon quality. However, patients with concurrent LHB tenodesis showed lesser fatty infiltration. No study has examined the radiological outcome between patients with rotator cuff repair without concomitant tenodesis of the LHB and those with no LHB surgery to date. The vast majority of studies conducted on LHB have compared tenodesis and tenotomy. In a study comparing LHB tenotomy to tenodesis with concomitant rotator cuff tear, ultrasonography 24 months postoperatively indicated 100% coverage of the rotator cuff footprint in both groups [30]. In their study on 209 patients 10 months after rotator cuff repair without LHB tenodesis, Lee et al. found an association between the deterioration of preserved LHB and greater fatty infiltration of the superior rotator cuff. In another study comparing LHB tenotomy with subpectoral tenodesis, the overall rate of rotator cuff tears on MRI was 20% (eight patients in the tenotomy group and 10 in the tenodesis group). The current results partially confirm the aforementioned studies’ findings. The study’s group with concomitant tenodesis of the LHB demonstrated a similar re-rupture rate as reported above. The current study confirms Lee et al.’s results regarding the association between LHB preservation and higher fatty infiltration, which was surprising since the researchers expected more pronounced fatty infiltration in the LHB tenodesis group. This is because repositioning the LHB insertion in the biceps groove through tenodesis can reduce the caudalizing effect of the LHB on the humeral head, so the humeral head should exert more traction on the reconstructed SSP. This can negatively influence the re-rupture rate and fatty infiltration of the SSP. Future researchers could examine the radiographic outcome of rotator cuff repair with LHB treatment compared to rotator cuff repair without it, focusing on fatty infiltration to clarify why patients who received LHB tenodesis tend to have less fatty infiltration.

Limitations

This study has several limitations. First, patients were not randomly assigned to specific groups in advance because the study used a retrospective design; randomization was therefore impossible a priori. Since pathologies of the LHB often cannot be identified on preoperative MRI, the decision to treat the LHB with tenodesis is often made intraoperatively. Performing a priori randomization of patients before surgery thus would improve the study design but would compromise the translationality of the results. To date, this limitation does not appear to have affected the relevance of this study’s results. Second, this study included patients with concomitant pathologies such as acromioclavicular arthrosis or partial tears of the subscapularis tendon. Isolated SSP tears with or without concomitant LHB involvement are rare in clinical practice; including patients with concomitant pathologies therefore reflect clinical practice. Concomitant pathologies and corresponding surgical procedures were compared between the two groups to exclude bias in this regard, and no difference was found. This limitation thus does not disqualify this study’s findings. Third, a limited number of patients was analyzed in this study because the selection criteria were selected as rigorously as possible. A sample size calculation was performed to minimize this bias and revealed a suitable power, which indicates that the study has sufficient validity. Fourth, the MRIs analyzed in this study were achieved with a 1.5 rather than a 3.0 Tesla MRI. A 3.0 Tesla MRI would have further improved the measurements’ accuracy due to the higher resolution; however, the researchers do not have a 3.0 Tesla MRI in their department, which reflects the situation in most hospitals and therefore is not a major limitation in the authors’ perception. Fifth, in this study, only one of the two observers repeated the measurements after six weeks; nevertheless, both observers were trained to make the measurements a priori. Finally, the repair techniques used to treat the included patients were not identical because surgical treatment of rotator cuff tears often depends on the tear morphology assessed during the operation. This limitation is important and probably biased the results. However, most of the studies that have addressed this topic could not guarantee identical surgical treatments. So far, this limitation has not had an impact on this study’s findings.

## Conclusions

In the present study, no difference in clinical outcome between patients with rotator cuff repair without LHB surgery and those with concomitant LHB tenodesis at two years follow-up was found. In addition, we observed no difference in the re-rupture rate between both groups. Concerning tendon quality this study showed that patients with rotator cuff repair with concomitant LHB tenodesis present less fatty infiltration compared to patients with rotator cuff repair without LHB surgery. Concomitant LHB tenodesis, if indicated, seems to not have a detrimental effect on the clinical or radiological results but is associated with less fatty infiltration than rotator cuff repair without LHB surgery. Further studies with a longer follow-up period are required to verify the results of the present study.
